# Primary Diffuse Large B-Cell Lymphoma of Central Nervous System: Is Still Surgery an Unorthodox Treatment?

**DOI:** 10.14740/jocmr2376w

**Published:** 2015-10-23

**Authors:** Ioannis Siasios, Aggeliki Fotiadou, George Fotakopoulos, Maria Ioannou, Vassilios Anagnostopoulos, Konstantinos Fountas

**Affiliations:** a Department of Neurosurgery, University Hospital of Larissa, Mezourlo 1, Larissa 41110, Greece; b Department of Pathology, University Hospital of Larissa, Mezourlo 1, Larissa 41110, Greece

**Keywords:** Primary CNS lymphoma, Diffuse large B-cell lymphoma, Radiotherapy, Chemotherapy

## Abstract

Primary central nervous system lymphoma (PCNSL) is characterized as an extra-nodal non-Hodgkin lymphoma which develops from the neuraxis. The purpose was to report a case of a patient with a supra-tentorial tumor who underwent subtotal resection of his tumor as his biopsy was not indicative of a PCNSL tumor and had uneventful recovery until his last follow-up. A 42-year-old man was admitted to our department for generalized epileptic seizures. CT and MRI examinations revealed a tumor in his right parietal-occipital lobe that was surrounded by edema and was enhancing after gadolinium administration. The patient underwent a navigation-assisted parieto-occipital craniotomy and posterior parietal transcortical approach for tumor biopsy which was not indicative of PCNSL tumor. The surgical team decided to remove the tumor on site. Histological analysis of the resected specimen showed primary diffuse large B-cell lymphoma. Combined chemotherapy and radiation therapy was applied to the patient, and at his last follow-up (16 months), he is tumor free. In our case as in several other studies during the last decade, the outcome after the surgical resection of a PCNSL tumor in combination to radiation and chemotherapy was unexpectedly good. The role of surgery probably should be reconsidered for single lesion PCNSL tumors.

## Introduction

Primary central nervous system lymphomas (PCNSLs) are non-Hodgkin lymphomas that are developing outside the nodules and consist only the 3-4% of primary brain tumors [1-4]. Bailey first described PCNSL as “perithelial sarcoma” of the CNS and Henry in 1974 recognized its lymphoid origin [3]. The majority of these tumors (95%) are considered diffuse large B-cell lymphomas (DLBCLs) [5, 6]. DLBCLs are characterized as high grade and most of them are CD20 positive while the minority of PCNSL consists of types such as Burkitt lymphomas, Burkitt-like lymphomas and lymphoblastic lymphomas [1, 7, 8].

PCNSL typically develops in the fifth to seventh decade of life although its overall incidence has increased in the last decade probably due to technological advances that contribute significantly in the earlier diagnosis [9]. It can be present in the ocular region, spinal cord and CSF [7, 10]. Supratentorial single lesions occur in about 60-70% of the patients [11].

Headache, nausea, vomiting, epileptic seizures, disorders of speech and vision and focal neurologic deficits are the most common symptoms of brain lymphomas. Diagnosis requires computer tomography (CT) of the brain, magnetic resonance imaging (MRI), microscopic ocular examination, CSF analysis and biopsy [7]. In case that the biopsy is received from the infiltrative margins of the tumor, PCNSL resembles morphologically to glial tumors, especially oligodendroglioma. Their occurrence is associated with situations involving immune deficiency, such as organ transplantations and HIV [7, 11, 12].

The recommended treatment for these patients includes chemotherapy with or without additional radiotherapy, whereas corticosteroids can be used in addition. Surgery is not recommended for the treatment of brain lymphomas and it is only performed in extreme situations [7]. Generally, PCNSL of DLBCL type is a highly aggressive tumor and although its prognosis is poor, about one-third of younger patients can hope for cure of the disease [13]. In this short communication, authors are presenting a case of a DLBCL tumor which was treated surgically and had an unexpected good outcome.

## Case Report

A 42-year-old right-handed man was referred to our neurosurgery department for generalized epileptic seizures, muscular weakness of both upper and lower limbs, gait instability and headache. The patient was suffering from recurrent seizures since his childhood and was under anticonvulsant treatment with phenobarbital and valproic acid. He was a heavy smoker and had a history of dyslipidemia and allergy in amoxicillin. Also, in the past, the patient underwent a surgery on his right hip after a traffic accident that he had 2 years before.

**Figure 1:**
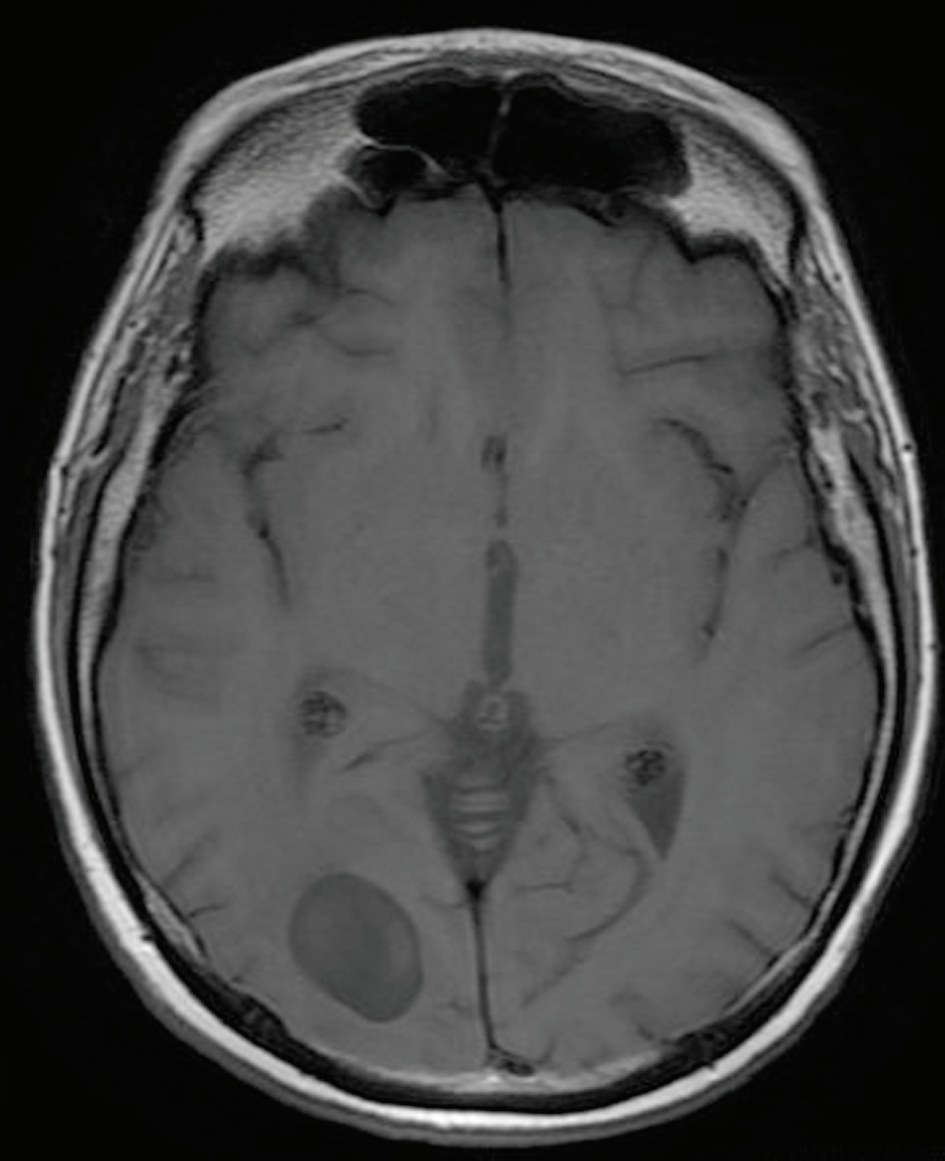
Pre-operative brain MRI T1 sequence.

**Figure 2:**
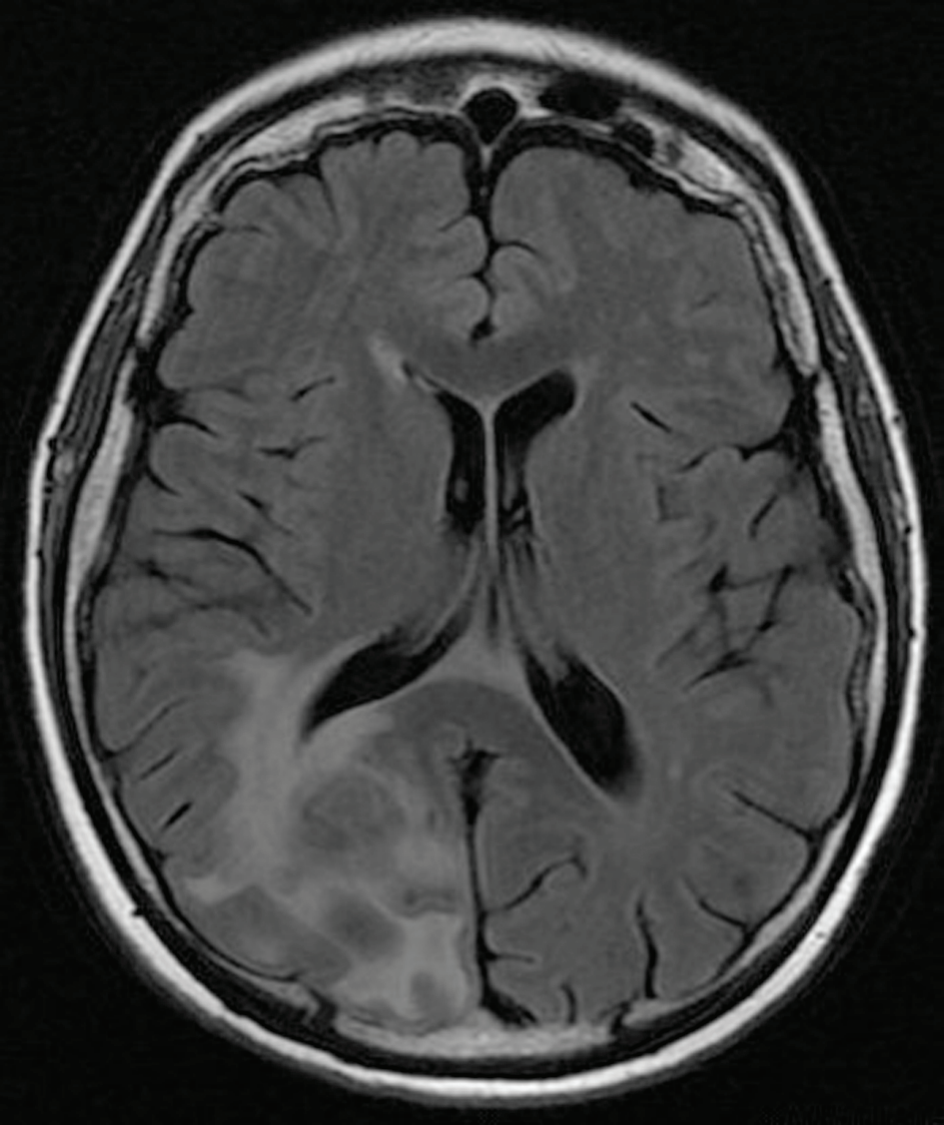
Pre-operative brain MRI FLAIR sequence which shows the peritumoral edema.

**Figure 3:**
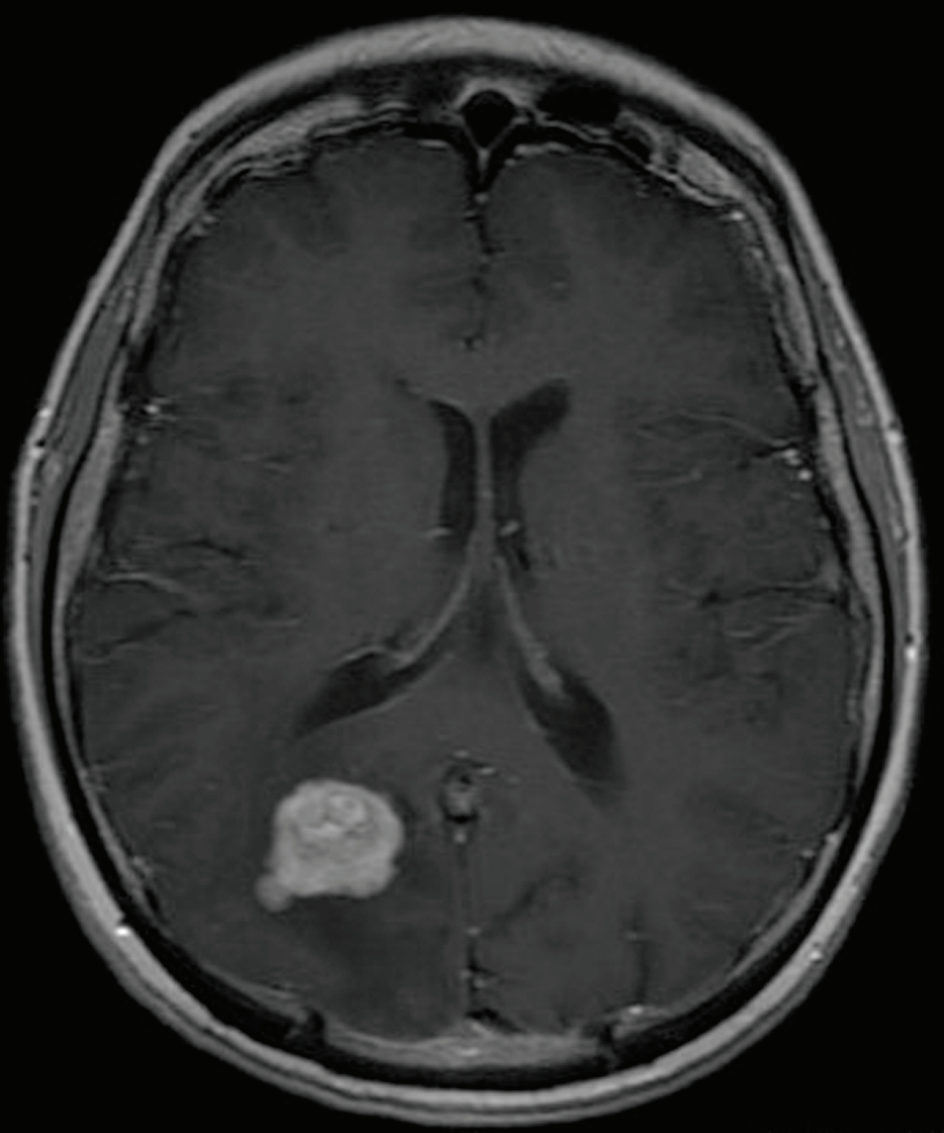
Pre-operative brain MRI T1 sequence with contrast gadolinium which shows the enhancing of the tumor.

**Figure 4:**
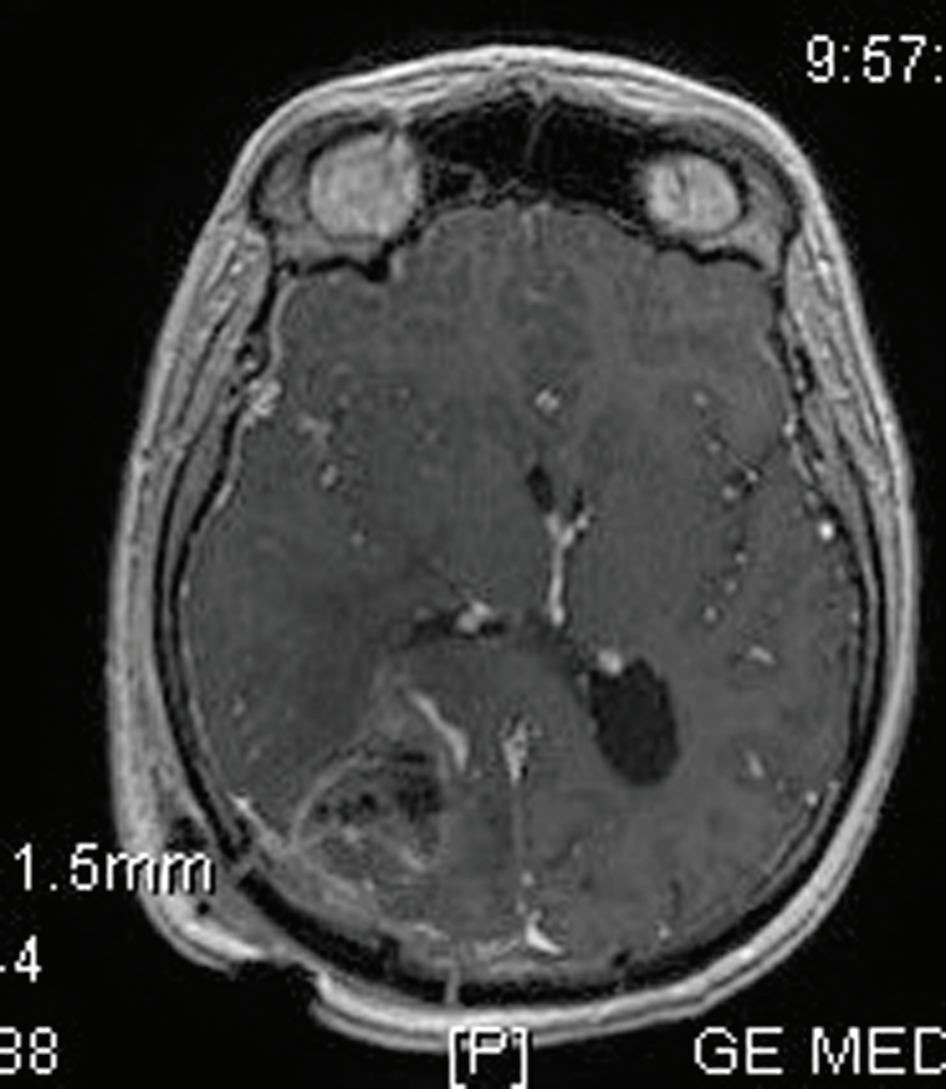
Post-operative brain MRI SPGR imaging which confirms the removal of the tumor.

**Figure 5:**
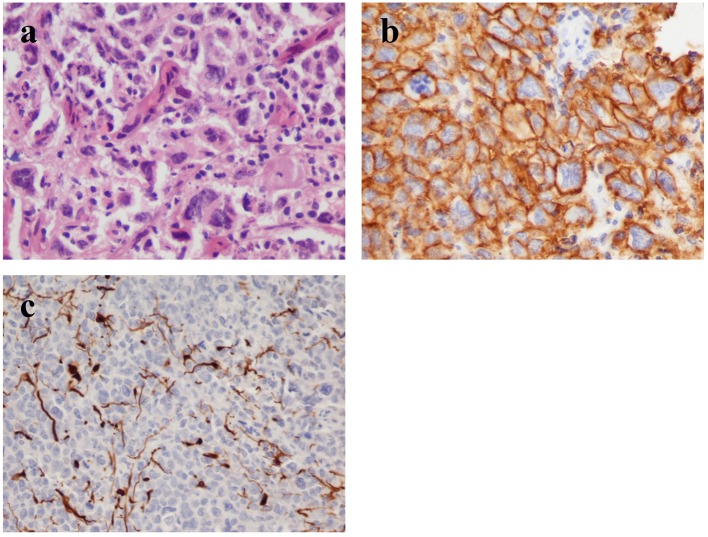
(a) Histology shows perivascular spread of highly anaplastic lymphoid cells with numerous mitotic figures (hematoxylin and eosin stain, original magnification × 40). (b) The tumor cells express the pan-B-cell marker CD20 (immunohistochemistry, original magnification × 40). (c) The malignant cells invade the parenchyma which demonstrates glial fibrillary-acid protein (GFAP) immunoreactivity (immunohistochemistry, original 15 magnification × 20).

His clinical examination showed increased reflexes of his left leg without other neurological findings. A brain CT scan without contrast was initially performed and revealed a lesion in his right parietal-occipital lobe. The additional intravenous administration of contrast medium showed that the dimensions of the lesion were 7.5 × 2 × 4.6 cm. The contrast uptake and the density of the tumor were heterogeneous. There was also 1 cm middle line shift to the left as well as elevation of the right occipital horn. The patient had screws in his right hip, which were surgically removed in order to perform an MRI for further investigation of the tumor. MRI examination confirmed the existence of a lesion in the right occipital lobe, nearby the occipital horn compatible with the image of a low grade glioma (Fig. 1-3). The patient did not have any visual disorders as the opthalmologists reported before surgery. Also, blood exams did not reveal any numerical or functional disorders. The CT of the thorax, pelvis, upper and lower abdomen did not show any other pathological signs. The patient was scheduled for an occipital craniotomy for biopsy and depending on the result, resection or not of the lesion. Due to the inability of the biopsy to clarify the type of the tumor and considering the young age of the patient, we proceeded to surgical excision of the tumor. Intraoperative neuromonitoring (motorsensory and visual evoked potentials) and neuronavigation were used. The tumor was resected with the use of microscope and CUSA. A post-surgical MRI was performed after the operation and confirmed the subtotal excision of lesion (Fig. 4). CSF analysis did not reveal any pathological findings. Histopathological analysis revealed DLBCL (Fig. 5a-c) and the patient was referred to hematologists-oncologists of our hospital for further treatment manipulations. The patient was treated with radiotherapy and methotrexate and 16 months after his surgery is still tumor free.

## Discussion

PCNSLs represent 0.7-1.7% of all malignant tumors and less than 5% of tumors arising within skull and spine [7, 14]. PCNSLs occur in more than 50% of cases in supra-tentorial areas [7, 15]. PCNSLs are mainly DLBCL, thereby they are characterized by aggressiveness [15, 16]. Diagnosis is established 3 - 6 months after the symptoms onset [7]. Patients have cognitive deficits, psychomotor slowing and mental alteration. Approximately 50% of cases have focal symptoms, headaches and increased intracranial pressure, while cranial nerve disorders and cerebellum or brainstem symptoms are present to the minority [15]. Differential diagnosis includes metastasis, glioma and toxoplasmosis, whereas in rare cases PCNSLs have signs of demyelinating disease [7].

Generally surgical excision should be avoided because these tumors are often cited in deep locations and therefore the risk of post-operative neurologic complications is increased [17, 18]. Furthermore, several studies suggest that resection provides no benefit in survival of patients with PCNSL [12, 17]. Thus, surgical interventions are restricted to a stereotactic biopsy for diagnostic purposes only [17, 19]. It is important that the patient is not medicated with corticosteroid agents before biopsy takes place [15, 20]. Corticosteroids can relieve the brain from the peritumoral edema, but they induce apoptosis of the tumor cells and therefore their administration prior to biopsy may preclude diagnosis [15, 21]. In our case, the patient was under corticosteroid treatment before surgery and this may be a significant factor for the failure of identification in the biopsy. In the histopathologic examination, the tumor cells express CD19, CD20 and CD79a, whereas the majority expresses Bcl-6 and MUM1 and in some cases CD10 is expressed as well. The high mitotic activity leads to the increase of the MIB1, which can exceed 90% [15]. Occasionally, necrosis is also noted in histopathologic analysis.

The disease has unfortunately poor prognosis and patients are expected to survive for 3 - 6 months without treatment, whereas chemotherapy alone or in combination with radiotherapy may improve median survival time to 25 - 60 months [7, 13]. It is apparent that younger patients (under the age of 60) and patients whose lymphoma does not affect meninges or the regions in proximity to ventricles have better prognosis [22]. The same applies to individuals whose immunity is normal [23]. Concentration of serum lactate dehydrogenase (LDH) is an independent prognostic marker [22]. Increased LDH and protein levels in CSF generally indicate poor prognosis [22].

Currently the standard treatment of PCNSL is high-dose methotrexate (HD-MTX)-based chemotherapy, which can be combined with whole-brain radiation (WBR) [24]. However, the employment of additional radiotherapy has been controversial due to its association with considerable neurotoxic effects [18, 25, 26]. It has been clearly indicated that the addition of WBR in first-line chemotherapy has a favorable role in progression-free survival (PFS) and some authors suggest that this also applies to overall survival (OS) [27-33]. Nevertheless, according to Thiel et al, the impact on OS is not significant and these results are also supported by several studies [27, 34, 35]. The omission of WBR from first-line treatment and its deferral until relapse of the disease may reduce treatment-related neurotoxicity and thus it is an acceptable and rational option for elderly patients, for whom the risk of neurologic impairment is higher compared to young patients [18, 24, 27-29, 36-38]. This approach however may not be the optimal solution for young patients, because the achievement of a prolonged PFS is a major therapeutic aim in this age population [17, 28, 39]. Moreover, Prica et al demonstrated that combined chemoradiotherapy significantly maximizes life expectancy and quality-adjusted life expectancy and proposed that this is the most suitable treatment for young patients [37]. Other therapeutic approaches that have been explored in order to diminish the neurotoxicity induced by additional WBR yielded quite promising results. These include low-dose WBR with tumor bed boost after chemotherapy [29], consolidation therapy with autologous stem-cell transplant [40] and pemetrexed as a single agent for elderly patients [41].

As previously mentioned, surgery is considered to have a limited role in the confrontation of PCNSL. However this notion is challenged in a secondary analysis of the G-PCNSL-SG-1 trial, where PCNSL patients who underwent tumor resection achieved improved OS and PFS compared to biopsied patients. The favorable impact of resection regarding OS was less significant after adjustment for the number of lesions. Authors concluded that tumor removal should be considered in the treatment of lesions amenable to resection, especially in cases of single lesions [42]. According to Nishikawa, if the results of this study are valid, then tumor resection may represent a better approach for selected PCNSL cases than biopsy alone [43]. In a retrospective analysis of 248 patients [12], the 1-year survival rate was higher in patients who underwent complete excision than in those who underwent stereotactic biopsy and although this result was not statistically significant, it could imply therapeutic benefit [19, 44]. Case reports have presented patients successfully treated with tumor excision. Sonstein et al reported a patient who survived over 5 years disease-free following complete tumor excision and a short course of steroid treatment [45]. Davies et al described a patient who survived over 20 years after total resection of his tumor [46]. Trapella et al presented a patient who remained tumor-free 79 months after total tumor removal and combined chemoradiotherapy [47]. Furthermore, in a study of 32 patients, Bellinzona et al concluded that surgery may be beneficial for patients with large-single space occupying lesions and progressive deterioration of neurologic status [48]. Tumor removal should also be considered in cases with the risk of imminent brain herniation [19]. Clinical experience of several researchers has shown the potential positive role of surgery in facilitation of glucocorticoid tapering, eradication of drug-resistant cell populations and alleviation of mass effect in cases of accessible, well-circumscribed lesions [49]. Previous studies that support the inferiority of surgery may no longer be sufficient to assess its curative role, because the technical advances in neurosurgery and the amelioration of adjuvant treatments have contributed to the efficiency and safety of surgical resections [42, 50]. Therefore, future studies should be conducted in order to evaluate extent of tumor removal in PCNSL patients [51].

### Conclusion

Although surgery is not the gold standard treatment for DLBCL tumors, presurgical imaging and clinical evidence in our case were not indicative for primary lymphoma. Also, this lesion was reachable for surgical excision as a single one with minimal danger for neurological deficits. The successful treatment management of our case may raise the question of early surgical excision in single primary lymphomas when they are located in non-eloquent areas. We concur that future prospective studies should be performed to further evaluate the capacity of surgery in the treatment of PCNSLs.

## Abbreviations

PCNSL: primary central nervous system lymphoma
DLBCL: diffuse large B-cell lymphoma
CNS: central nervous system
